# Effects of Intratympanic Steroid Therapy in Sudden Hearing Loss in Children: The Clinical Case of an Exceptionally Late Hearing Recovery

**DOI:** 10.22038/ijorl.2025.83020.3794

**Published:** 2025

**Authors:** Marco Capelli

**Affiliations:** *ENT Unit, Clinica Villa Antonella, Codogno (Lo), Italy. *

**Keywords:** Sudden hearing loss, Children, Childhood, Intratympanic steroid therapy, Profound hearing loss

## Abstract

**Introduction:**

Sudden hearing loss represents a medical emergency that can potentially have a highly negative impact on an individual's quality of life. In particular it can represent a serious concern in pediatric age, interfering cognitive and language development. Despite the seriousness of the problem, there is currently no consensus on diagnostic and therapeutic management methods, and the debate is still open.

**Case Report:**

We present the case of a 15-year-old child with a sudden right-sided hearing loss of profound degree and of idiopathic etiology. Initially he was treated with oral steroid treatment and subsequently to multiple sessions of hyperbaric oxygen therapy without satisfactory results. Several weeks after the onset of symptoms, the patient underwent a cycle of 3 intratympanic methylprednisolone injections with unexpected hearing recovery.

**Conclusions:**

Intratympanic steroid therapy could represent not only a life-saving therapeutic choice as it is currently considered, but also a first therapeutic choice, particularly in pediatric age, thanks to substantial absence of systemic side effects and the potential efficacy even after many weeks from the onset of symptoms.

## Introduction

Sudden sensorineural hearing loss (SSNHL) is a medical emergency that requires immediate medical treatment (1-3). It is defined as a hearing loss of at least 30 dB across three consecutive frequencies occurring within a 72-hour period (2,3).

In the pediatric population, profound SSNHL (> 80dB) is observed in 55% of cases. If not treated promptly, it can lead to serious sequelae in language acquisition and cognitive development, having a significant impact on family and social dynamics (4,5).

Alexander et al. reported that the incidence of sudden hearing loss in the pediatric population is only 20-30 cases per 100,000 inhabitants per year (6,7) However, as reported by Feng Jiao Li et al. the prevalence of SSNHL in pediatric population appears to be steadily increasing (8). 

In 2021, Wood et al. published a meta-analysis of 13 studies and 605 pediatric patients suffering from SSNHL suggesting that the association between hearing loss and Cytomeglovirus (CMV) infection is frequent (9). In the same year, Reading et al. published a review of the literature stating that although there are many possible causes of sudden hearing loss in children (infectious, autoimmune, vascular, hormonal), in most cases the etiology remains unknown and is defined as idiopathic (3). Furthermore, in 2024, the first cases of SSNHL in the pediatric population related to SARS Cov2 infection were reported (10) SARS Cov2 is known for its high neurotropism and significant neurotoxicity. These features have been widely supported and described since the first studies dating back to 2020. (11-13)

The SSNHL treatment in children is still very controversial today, there is no protocol to follow in these cases. Specifically, the role of steroid therapy is still to be clarified. It is believed that the majority of pediatric otolaryngologists treat SSNHL with systemic steroids (14), although intratympanic administration of the drug has also been proposed in many cases (3,9). According to some authors, however, there is no evidence to support intratympanic treatment (15).

We report the clinical case of a 15-year-old child suffering from sudden hearing loss resulting in complete right-sided anacusis which was partially recovered only after intratympanic steroid therapy although carried out many months after the onset of symptoms. The parents of the patient, a minor, provided informed consent for the publication of their medical information. 

## Case Report

On 12-04-24 M.R., male, Caucasian, 15-year-old, reported the sudden onset of right-sided hearing was associated with ipsilateral tinnitus. He did not complain of ear pain or dizziness. He had no fever. There were no symptoms of nasal obstruction or rhinorrhea, and he denied any history of trauma. 

The patient was in good general health conditions. No illnesses to report, no allergies. He doesn't take any medications.

On 19-04-24, given the persistence of the symptoms, he went to the hospital emergency room where he underwent a blood count which showed values ​​within normal limits and no other blood chemistry tests. He was Then evaluated by an otolaryngologist. He underwent otoscopy with evidence of minimal quantities of wax inside the external auditory canal, which was removed, without improvement in symptoms. 

**Fig 1:**
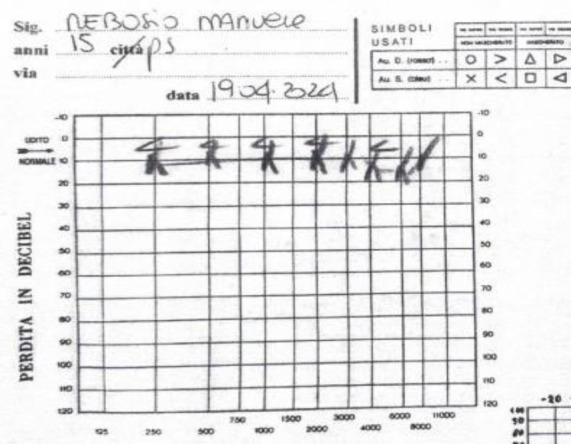
Audiogram performed on 19-04-24, at the time of diagnosis with evidence of right anacusis

At that time, underwent an audiometric examination which revealed left normoacusis and right anacusis (Fig. 1). The diagnosis of right-sided hearing loss was therefore established.

After programming a Nuclear Magnetic Resonance of the brain, the patient was discharged from the emergency room with oral steroid therapy according to the following scheme: Prednisone 50 mg per day for 4 days, then 25 mg per day for another 4 days and then 12.5 mg per day for another 4 days. 

**Fig 2:**
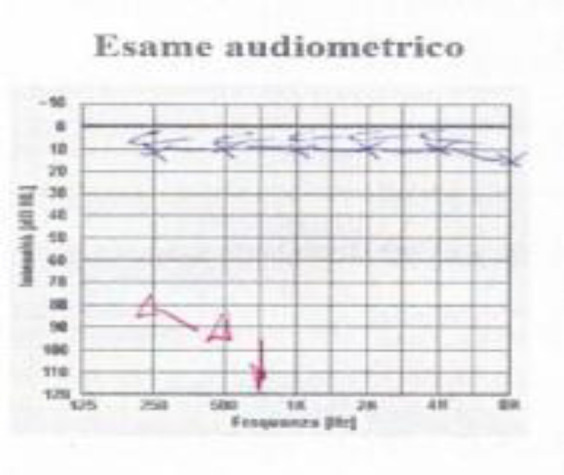
Audiogram performed on 02-05-24, after hyperbaric therapy, with evidence of minimal hearing recovery on low frequencies. The hearing threshold will remain unchanged at the tonal audiometric test performed at the end of the hyperbaric therapy on 10-06-24

Nuclear Magnetic Resonance was carried out on 02-05-24. Sections were performed according to the three orthogonal planes in space, weighted in T1, T2, Flair sequence, in diffusion and in gradient echo. Thin sections were performed for detailed evaluation of the posterior fossa structures and cranial nerves. The examination was completed with contrast medium. The result of the examination was normal. On 24-04-24 the patient began a cycle of hyperbaric therapy. A total of 32 sessions were performed, with a frequency of one session per day at a pressure of 2.5 ATA. Each therapy session lasted 90 minutes and was divided into 2, spaced phases, each with the supply of pure oxygen lasting 36 minutes. After 6 sessions, on 02-05-24, a control audiometric test was performed with evidence of a very slight recovery of the hearing threshold on low frequencies. Specifically, as per the graph in Fig. 2, the patient was able to perceive the 250 and 500 Hz frequencies at 80 and 90 dB respectively. No improvement was recorded on all the other frequencies tested (1000, 2000, 4000 and 8000 Hz). For this reason it was decided to continue the hyperbaric treatment with the aim of further improvement. On 10-06-24 the patient underwent the second audiometric check during hyperbaric therapy. Since the situation remained unchanged compared to the previous one, it was decided to conclude hyperbaric treatment. 

**Fig 3:**
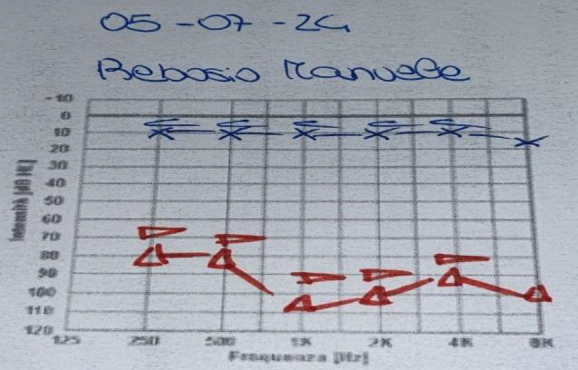
Audiogram performed on 05-07-24, after the first intratympanic injection, shows an initial recovery also on medium and high frequencies

**Fig 4:**
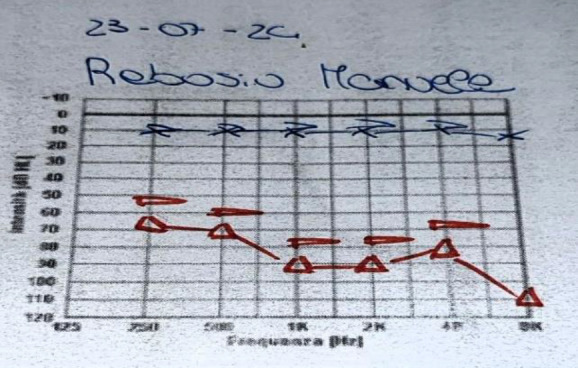
Audiogram performed on 23-07-24, after the second intratympanic injection, shows shows further recovery across all frequencies

On 28-06-24 the patient is evaluated at our clinic. We repeated a tonal audiometric test which remained unchanged compared to the last one performed. Speech audiometry could not be performed. Although there was minimal hearing recovery at low frequencies, the patient reported that he did not subjectively notice any significant hearing improvement after steroid and hyperbaric treatment. On same day the patient received the first intratympanic injection of Methylprednisolone under local anesthesia performed using lidocaine. The injection was performed with a 25 G spinal needle at the level of the antero-inferior quadrant. 0.9 ml of Methylprednisolone (to the concentration of 40 mg/ml) was administered. The patient was monitored in the operating room for 30 minutes during which he lay supine with his head turned to the left, instructed not to speak or swallow. This procedure was repeated on 05-07-24 and on 23-07-24 with the same methods. Control tone audiometric examination was performed before each injection and after each of these the hearing threshold showed constant improvements as can be seen from Fig. 3 and Fig. 4. 

**Fig 5:**
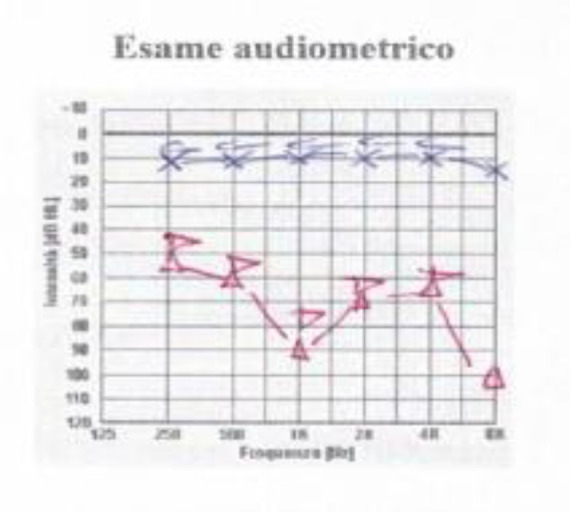
Audiogram performed on 06-08-24, after the third intratympanic injection, shows significant hearing recovery across all frequencies

The patient reported having noticed a gradual improvement in hearing quality already after the first injection, with a progressive and consistent benefit the second and third treatment. On 06-08-24 we carried out a check-up an optical microscope. The tympanic membrane appeared perfectly healed without residual perforations. We also performed the final audiometric exam that shows a clear hearing recovery on all tested frequencies (Fig.5).

We would like to point out that the patient did not take any additional drugs during the period of intratympanic therapy (from 28-06 to 06-08). He was able to regularly carry out normal daily activities including sports. During the treatment period the patient went to the seaside for a holiday during which he was advised to avoid to immerse his head below the water level and to dive. We also recommended that the patient avoid blowing his nose for a few days after the injections in order to reduce the risk of permanent perforation of the tympanic membrane. We did not observe any significant problems during or following the treatment nor did the patient report any particular issue to us.

## Discussion

Sudden sensorineural hearing loss represents an important medical emergency that can significantly affect the quality of life, especially in a delicate phase of life such as childhood and adolescence. However, regarding this condition with specific reference to the pediatric population, it is necessary to underline that there are no definitive guidelines for its management. Although we have treatment protocols for the adult population, we don’t have safe directions in childhood. There are some meta-analyses and reviews of the literature but none of these highlights certain data regarding the choice and modality of therapy (3,9,15-18).

The clinical case presented by us, despite being a single case and therefore devoid of statistical significance, could be useful as it leads us to reflect on two very important aspects about management of SSNHL in children that partly contradict with some data that emerge from the main works available in literature.

The first point concerns the significance of prognostic factors. According to the main reviews and meta-analyses currently available, there is general consensus among the authors regarding the consideration of early treatment as a positive prognostic factor (5,7,9). Furthermore, Wood et al, in their meta-analysis (9), states, in agreement with other authors (19) that the severity of hearing loss represents a negative prognostic factor. The hearing recovery that occurred in our patient suffering from anacusis, who did not respond to systemic steroid treatments and hyperbaric oxygen therapy and underwent intratympanic steroid treatment many weeks (11) after the onset of symptoms, seems therefore truly exceptional.

The second aspect on highlighted by this clinical case concerns the type of therapeutic choice. In the meta-analysis proposed by Franz et al. in 2021 the results obtained in children treated with systemic steroids are compared with those treated in a combined manner with systemic steroid-intratympanic steroids. The study concluded that there is a lack of evidence supporting the usefulness of intratympanic steroid treatment. 

Jacobs et al., in 2021, published an in-depth meta-analysis in which they addressed the topic of therapeutic choice. In this study, the authors acknowledge a significant role for intratympanic steroid therapy when performed after the failure of other treatments. 

However, they state that although there are multiple strategies of treatment for SSNHL in children, it is not currently possible to carry out an adequate comparison between them (3) to establish the most effective one. Others studies support usefulness of intratympanic steroid therapy (4, 19), Ovet et al (20), proposes a simultaneous intratympanic treatment in combination with systemic steroid, Pitaro et al. (21) reserves a rescue role for intratympanic therapy, carrying it out after systemic steroid treatment. 

In the case reported, in agreement with the latter and other authors (3), intratympanic steroid therapy was performed after the failure of systemic therapies. Based on our experience, we currently consider intratympanic administration to be a valid rescue therapy, in some cases very effective. 

Given what has been stated so far, including the absence of certain evidence and absolute indications regarding its administration and given the potential difficulty in carrying it out in pediatric population (22) we do not believe it should be considered the first-line treatment to be carried out in case of sudden sensorineural hearing loss, however we maintain that it should be considered a serious therapeutic option in cases of failure of systemic treatments.

## Conclusions

Sudden hearing loss is a medical emergency with particularly significant implications for affected patients, particularly in pediatric age (in the pediatric population). Currently, there are not definitive diagnostic and therapeutic pathways to follow for children and many questions remain open on this topic. 

The case presented contributes to this ongoing uncertainty, while also highlighting the potential of intratympanic steroid therapy, a treatment approach that is not yet fully recognized in clinical guidelines. Its substantial absence of systemic side effects and its possible efficacy even at a considerable distance from the onset of symptoms could represent, if supported by further numerically significant studies, an effective tool in the treatment of sudden hearing loss in children.

## References

[1] Lamounier P, Franco Gonçalves V, Ramos HVL, Gobbo DA, Teixeira RP, Dos Reis PC, Bahmad F Jr, Cândido Costa C (2020 ). A 67-Year-Old Woman with Sudden Hearing Loss Associated with SARS-CoV-2 Infection. Am J Case Rep.

[2] Skarżyńska MB, Kołodziejak A, Gos E, Sanfis MD, Skarżyński PH (2022). Effectiveness of Various Treatments for Sudden Sensorineural Hearing Loss-A Retrospective Study. Life (Basel)..

[3] Reading JCS, Hall A, Nash R (2021). Paediatric Sudden Sensorineural Hearing Loss: Pooled Analysis and Systematic Review. J Int Adv Otol..

[4] Qian Y, Zhong S, Hu G, Kang H, Wang L, Lei Y (2018). Sudden Sensorineural Hearing Loss in Children: A Report of 75 Cases. Otol Neurotol..

[5] Xiao L, Su S, Liang J, Jiang Y, Shu Y, Yao H, Ding L (2022 ). Clinical features and prognostic factors of children with profound sudden sensorineural hearing loss. Front Pediatr.

[6] Alexander TH, Harris JP (2013 ). Incidence of sudden sensorineural hearing loss. Otol Neurotol.

[7] Chung JH, Cho SH, Jeong JH, Park CW, Lee SH (2015 ). Multivariate analysis of prognostic factors for idiopathic sudden sensorineural hearing loss in children. Laryngoscope.

[8] Li FJ, Wang DY, Wang HY, Wang L, Yang FB, Lan L, Guan J, Yin ZF, Rosenhall U, Yu L, Hellstrom S, Xue XJ, Duan ML, Wang QJ (2016). Clinical Study on 136 Children with Sudden Sensorineural Hearing Loss. Chin Med J (Engl)..

[9] Wood JW, Shaffer AD, Kitsko D, Chi DH (2021). Sudden Sensorineural Hearing Loss in Children-Management and Outcomes: A Meta-analysis. Laryngoscope..

[10] Chrysouli K, Savva IP, Karamagkiolas S (2024). The First Cases of Sudden Sensorineural Hearing Loss Post Coronavirus Disease in Children. J Audiol Otol..

[11] Capell M, Gatti P (2020). Anosmia and COVID-19 in south Lombardy: description of the first cases series in Europe. B-ENT.

[12] Capelli M, Gatti P (2021). Anosmia in the first coronavirus disease 2019 outbreak in Europe: functional recovery after eight months. The Journal of Laryngology & Otology..

[13] Gerstacker K, Speck I, Riemann S, Aschendorff A, Knopf A, Arndt S (2021). Deafness after COVID-19?. HNO.

[14] Luu K, Shaffer AD, Chi DH (2023). Practice trends in pediatric sudden sensorineural hearing loss management: An unresolved diagnosis. Am J Otolaryngol..

[15] Franz L, Gallo C, Marioni G, de Filippis C, Lovato A (2021). Idiopathic Sudden Sensorineural Hearing Loss in Children: A Systematic Review and Meta-analysis. Otolaryngol Head Neck Surg..

[16] Conlin AE, Parnes LS (2007). Treatment of sudden sensorineural hearing loss: I A systematic review. Arch Otolaryngol Head Neck Surg..

[17] Chandrasekhar SS, Tsai Do BS, Schwartz SR, Bontempo LJ, Faucett EA, Finestone SA, Hollingsworth DB, Kelley DM, Kmucha ST, Moonis G, Poling GL, Roberts JK, Stachler RJ, Zeitler DM, Corrigan MD, Nnacheta LC, Satterfield L, Monjur TM (2019). Clinical Practice Guideline: Sudden Hearing Loss (Update) Executive Summary. Otolaryngol Head Neck Surg..

[18] Fetterman BL, Saunders JE, Luxford WM (1996). Prognosis and treatment of sudden sensorineural hearing loss. Am J Otol..

[19] Chen YS, Emmerling O, Ilgner J, Westhofen M (2005). Idiopathic sudden sensorineural hearing loss in children. Int J Pediatr Otorhinolaryngol..

[20] Övet G, Alataş N, Güzelkara F (2016). Sudden Pediatric Hearing Loss: Comparing the Results of Combined Treatment (Intratympanic Dexamethasone and Systemic Steroids) With Systemic Steroid Treatment Alone. Otol Neurotol..

[21] Pitaro J, Bechor-Fellner A, Gavriel H, Marom T, Eviatar E (2016 ). Sudden sensorineural hearing loss in children: Etiology, management, and outcome. Int J Pediatr Otorhinolaryngol.

[22] Dedhia K, Chi DH (2016 ). Pediatric sudden sensorineural hearing loss: Etiology, diagnosis and treatment in 20 children. Int J Pediatr Otorhinolaryngol.

